# A Prospective Study Comparing the Efficacy of Hyaluronic Acid and Collagen in the Treatment of Nasolabial Folds

**DOI:** 10.1111/jocd.70630

**Published:** 2025-12-26

**Authors:** Chen Huang, Jiaxi Liu, Lei Guo, Qing Liu, Baoqiang Song

**Affiliations:** ^1^ Department of Plastic Surgery Xijing Hospital, Fourth Military Medical University Xi'an China; ^2^ Department of Dermatology The Second Affiliated Hospital of Kunming Medical University Kunming China; ^3^ Xian Bravou Medical Beauty Hospital Xi'an China

**Keywords:** collagen, hyaluronic acid, nasolabial fold

## Abstract

**Background:**

Hyaluronic acid (HA) is established as the gold standard for nasolabial folds (NLFs) correction. However, its use is frequently associated with transient edema and potential migration. These limitations have spurred investigation into collagen‐based fillers as viable alternatives characterized by comparable efficacy and a favorable safety profile.

**Objective:**

This prospective randomized controlled trial aimed to evaluate the comparative effectiveness of HA and collagen fillers in NLFs augmentation.

**Methods:**

Between January 2022 and December 2023, 100 patients with moderate to severe NLFs (Wrinkle Severity Rating Scale Grades 2–4) were enrolled and randomly allocated to receive either collagen (*n* = 50) or HA (*n* = 50) injections. Treatment protocols employed tailored injection doses according to baseline NLFs severity and indentation depth. Global Aesthetic Improvement Scale (GAIS) evaluation of standardized photographs was performed to assess outcomes at baseline, 3 months, and 6 months.

**Results:**

Baseline characteristics showed no intergroup disparities. Both materials produced similar improvements in NLFs reduction initially and at 3 months. At 6 months, HA maintained significantly greater improvements in NLF dimensions compared with collagen. GAIS scores indicated superior immediate and 3‐month aesthetic outcomes with collagen, while HA showed better aesthetic maintenance at 6 months.

**Conclusion:**

This study demonstrated comparable short‐term efficacy between collagen and HA for NLFs correction. These findings suggest collagen may be a practical alternative for patients seeking rapid correction with minimal downtime, while HA remains superior for long‐term correction. Treatment selection should therefore be individualized based on patient preference for immediate improvement or longevity.

**Trial Registration:**

Clinical Trial Registry: ChiCTR2500106800

## 1 Introduction

Extending from nasal ala to mouth corners, the NLFs enhance facial aesthetics by defining the cheek‐to‐lip contour and contributing to expression [1, 2]. With aging, the NLFs typically deepen and elongate, leading to significant aesthetic concerns [3]. Soft tissue fillers provide minimally invasive, immediate rejuvenation with low downtime [4], with HA fillers leading due to biocompatibility and durability (millions of U.S. procedures annually) [5, 6]. However, drawbacks like swelling, nodules, displacement, and discoloration persist, particularly in the dynamic NLFs area [7].

These limitations prompt exploration of alternatives like collagen‐based fillers, which offer strong biocompatibility and tissue integration but remain understudied owing to historical allergy risks and HA's market dominance. To date, no prospective randomized controlled trial has directly compared collagen and HA fillers specifically for NLFs correction. It is this critical knowledge gap that motivates the present study—a prospective, randomized controlled trial designed to rigorously compare the efficacy and safety of collagen versus HA fillers for NLFs correction.

## 2 Patients and Methods

This study was a parallel‐group, single‐blind randomized controlled trial. This study adheres to the Declaration of Helsinki and has received approval from the ethics committee of the local hospital. All patients provided informed consent. From January 2022 to December 2023, a total of 100 patients were included in the study. The inclusion criteria were as follows: (1) patients aged over 18 years; (2) an NLFs score of ≥ 2; (3) voluntary signing of the informed consent form. The exclusion criteria were: (1) the presence of midfacial diseases or other chronic conditions unsuitable for NLFs injection; (2) patients who had received facial treatments within the past 24 months; (3) patients who withdrew from the study midway.

### 2.1 Randomization and Blinding

**FIGURE 1:**
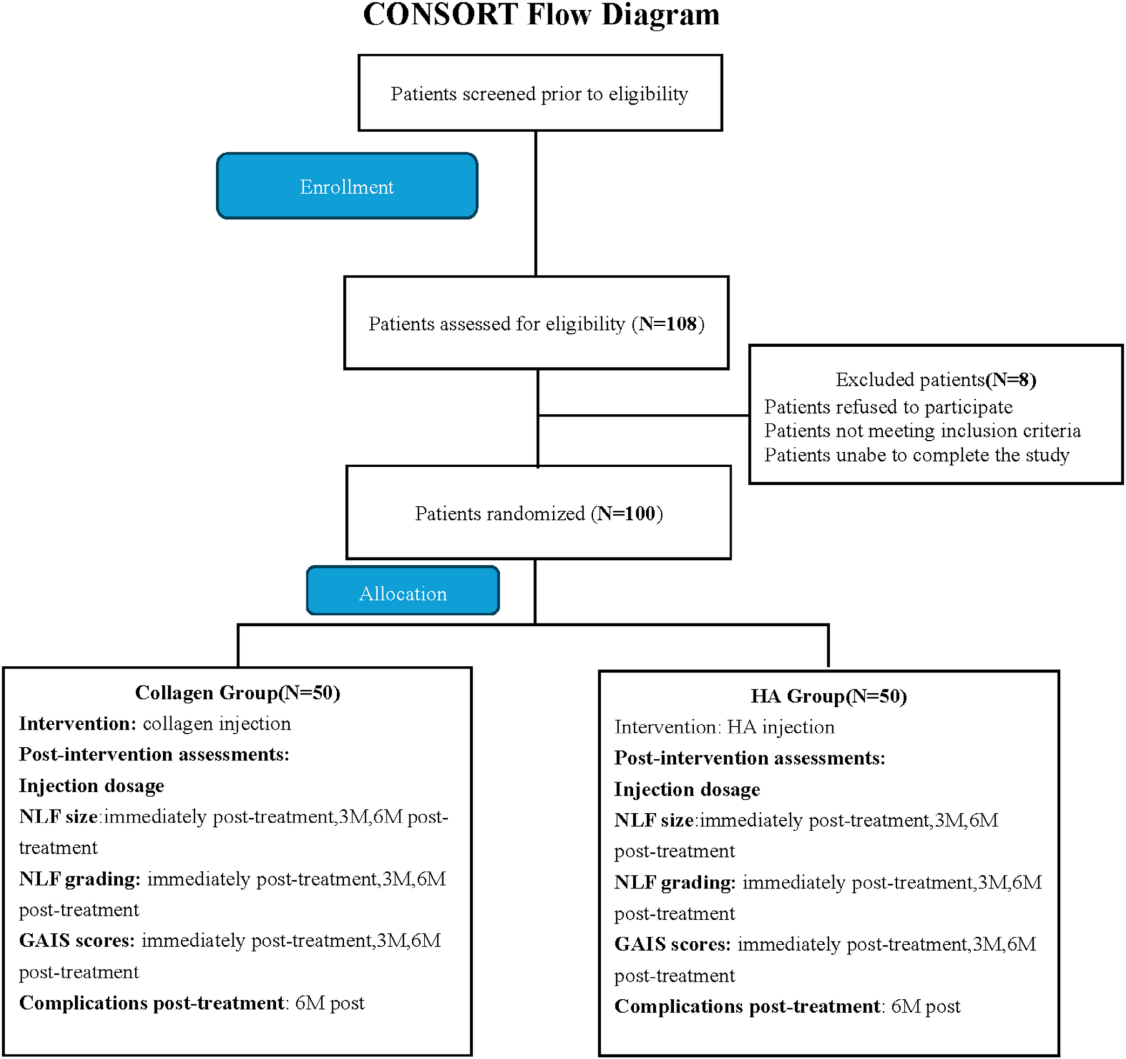
CONSORT flow diagram.

We assumed a Cohen's *d* of 0.5 for the NLFs improvement between groups, with *α* = 0.05 and power = 0.80. Accounting for a 10% attrition rate, a total of 50 patients per group were determined. This study employed a single‐blind, randomized controlled design. Patient randomization was performed using a random number table: an independent research coordinator generated the random sequence, assigning patient numbers (from 1 to 100) to either the experimental group (collagen) or the control group (HA). Allocation concealment was achieved through the use of opaque, sealed envelopes, each containing the patient's group assignment, which were opened by the injecting surgeon after patient enrollment. Blinding was implemented as follows: patients were unaware of the type of filler they received (patient blinding); the injecting surgeon was aware of the group assignment (as material selection was required, blinding was not feasible); outcome assessment was performed by another independent surgeon who was blinded to the patient's group allocation (assessor blinding). This design minimized observer bias (Figure 1).

### 2.2 Injection Technique (Figure 2)

**FIGURE 2:**
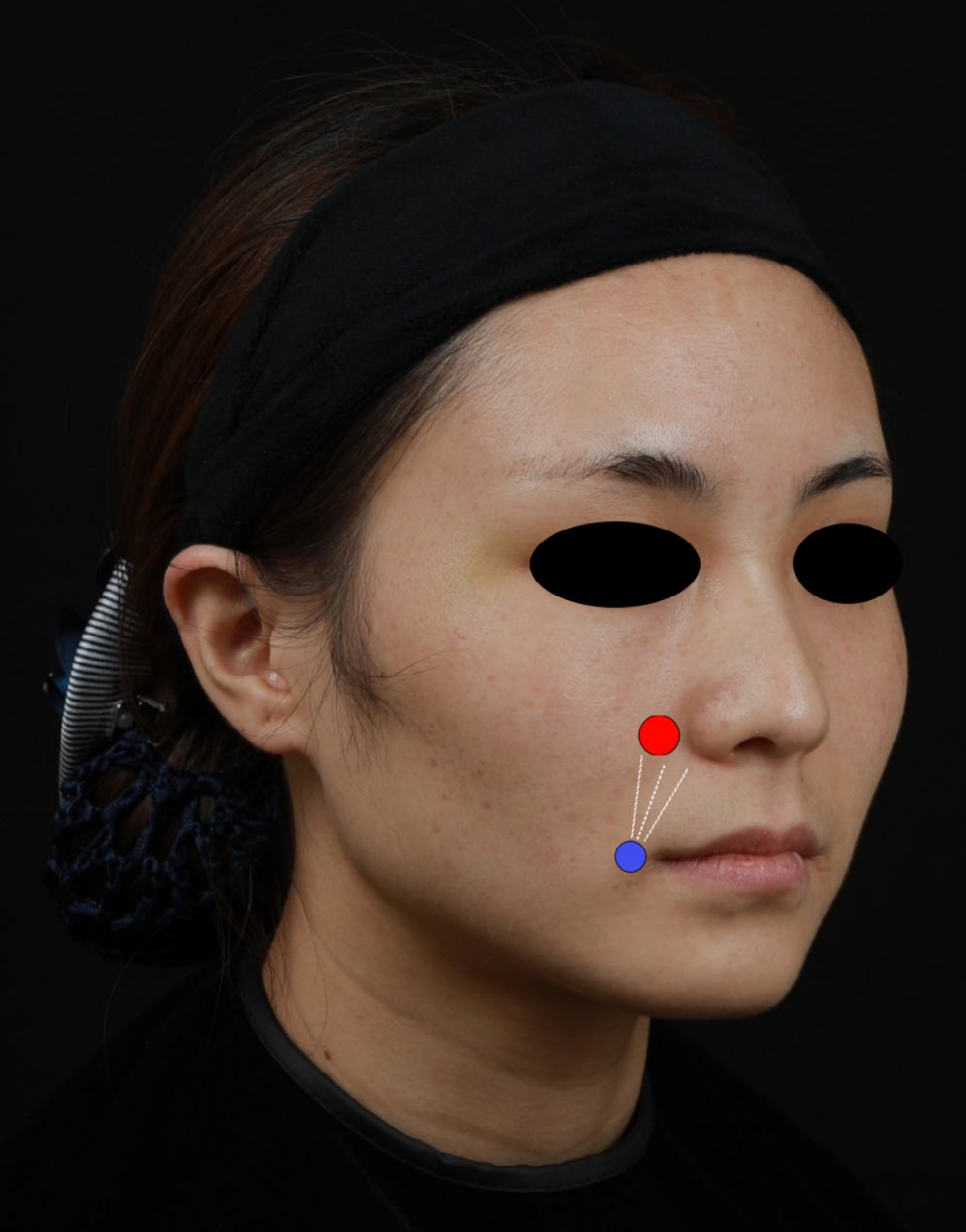
Illustration of nasolabial fold injection techniques: Collagen/HA fillers administered at the lateral pyriform aperture (red) via sharp needle vertical supraperiosteal deposition, and 1.0–1.5 cm lateral to oral commissure (blue) using blunt cannula linear fanning in subcutaneous fat plane.

#### 2.2.1 Injection Material Selection

Patients in the experimental group were treated with the collagen‐based filler Fillderm (Fuman Collagen; JBT Biotechnologies, Changchun, China) for NLFs correction. The control group received injections of the HA‐based filler Restylane Lyft (Galderma, Lausanne, Switzerland).

#### 2.2.2 Injection Technique

A dual‐layer technique was employed, targeting both the subcutaneous and periosteal layers.

#### 2.2.3 Subcutaneous Injection

A 27‐gauge blunt cannula was inserted 1–1.5 cm lateral to the oral commissure, with injection performed during withdrawal.

#### 2.2.4 Periosteal Injection

A 23‐gauge sharp needle was used for perpendicular injections at 2–3 predetermined points.

#### 2.2.5 Dosage Adjustment

The dose of HA or collagen was adjusted based on the patient's baseline NLFs severity grade.

#### 2.2.6 Post‐Injection Management

Gentle massage was applied to mold the product, followed by the application of a topical antibiotic ointment to prevent infection.

### 2.3 Assessment Item

The assessed indicators included: (1) baseline characteristics of both groups, such as age, gender, NLFs grading [8] (where 0 indicates no NLFs, 1 indicates a slight crease that is barely noticeable, 2 indicates shallow creases, 3 indicates moderate creases, 4 indicates clearly defined deep creases, and 5 indicates extremely deep and multiple creases), dimensions (including the length, width, and depth of the NLFs), and injection dosage; (2) comparison of NLFs size improvement immediately after treatment, at 3 months, and at 6 months between the two groups (NLFs dimensions were measured on the patient's face by an independent, blinded assessing surgeon. All measurements were performed at baseline, immediately post‐treatment, and at the 3‐ and 6‐month follow‐ups to ensure consistency. As the study was designed as a single‐assessor model and did not involve multiple assessors, inter‐rater reliability was not calculated.) This was the primary outcome of the study; (3) changes in NLFs grading immediately after treatment, at 3 months, and at 6 months between the two groups, this constituted the secondary outcomes of the study; (4) comparison of the Global Aesthetic Improvement Scale (GAIS) [9] scores between the two groups immediately after treatment, at 3 months, and at 6 months. The GAIS scoring criteria were as follows: −1 indicates deterioration, 0 indicates no change, 1 indicates slight improvement, 2 indicates noticeable improvement with room for adjustment, and 3 indicates complete improvement. These prospective assessments occurred at baseline, immediately post‐treatment, and at 3 and 6 months under standardized conditions to reduce bias. (5) complications post‐injection, these were the study's secondary outcomes. The study's success criterion was an NLFs grade improvement of ≥ 1, maintained for the filler's effective duration.

### 2.4 Statistic Analysis

This study adhered to the intention‐to‐treat principle for analysis. All enrolled patients were included in the final analysis, regardless of their completion of the follow‐up protocol. Regarding missing data management: since no patients withdrew or were lost to follow‐up in this study (all 100 patients completed the 6‐month assessment), no data were missing. SPSS 26.0 (IBM Statistics for Windows, IBM Corp., Armonk, NY, USA) was used for data analysis. Data were expressed as means ± standard deviations for continuous variables and medians for ordinal variables. Normality was assessed via Shapiro–Wilk test; since all data did not follow a normal distribution, non‐parametric tests (Mann–Whitney U test) were used for comparisons. A *p*‐value < 0.05 was considered statistically significant. Following this protocol, a standardized approach to patient injection and evaluation was implemented, and the results are presented below.

## 3 Results

### 3.1 Patient Demographics and Baseline Characteristics

From January 2022 to December 2023, 100 patients were enrolled and randomized, resulting in a complete dataset for analysis. The collagen and HA groups were well‐matched at baseline:

Demographics: The collagen group (*n* = 50, 37F/13M) had a mean age of 39.80 years, comparable to the HA group (*n* = 50, 42F/8M, mean age 41.25 years).

NLFs severity: Both groups had a median pre‐treatment NLFs grade of 3.

NLFs dimensions: Pre‐treatment length, width, and depth showed no significant differences between groups.

Filler dosage: The average total dosage was similar between the collagen (2.65 mL) and HA (2.55 mL) groups.

All baseline characteristics were balanced, confirming the success of randomization (Table 1).

**TABLE 1:** Baseline characteristics of patients.

Variables	Experimental group	Control group	*p*
Age, years	39.80 ± 8.29	41.25 ± 8.64	0.678
Gender	37F 13M	42F 8M	0.602
Grade of nasolabial folds	3 (3, 4)	3 (3, 3.75)	0.947
Size of nasolabial folds, mm			
Length	21.95 ± 3.87	21.09 ± 3.94	0.529
Width	4.51 ± 0.91	4.49 ± 0.87	0.925
Depth	0.75 ± 0.48	0.66 ± 0.36	0.718
Volume of injection, mL	2.65 ± 1.46	2.55 ± 1.32	0.862

### 3.2 NLFs Dimensional Changes

Changes in NLFs length, width, and depth from baseline were measured at multiple time points (Table 2).

**TABLE 2:** Changes in NLFs dimensions over time.

	Experimental group	Control group	*p*	95% CI
Length				
M_1_–M_0_	4.71 ± 2.69	3.91 ± 2.00	0.221	(−0.14, 1.74)
M_3_–M_0_	3.62 ± 2.30	3.43 ± 1.75	0.820	(−0.62, 1.00)
M_6_–M_0_	1.68 ± 1.12	2.67 ± 1.49	0.017*	(−1.51, −0.47)
Width				
M_1_–M_0_	0.71 ± 0.40	0.67 ± 0.27	0.301	(−0.10, 0.18)
M_3_–M_0_	0.58 ± 0.35	0.59 ± 0.26	0.883	(−0.13, 0.11)
M_6_–M_0_	0.28 ± 0.18*	0.49 ± 0.21	0.004*	(−0.29, −0.13)
Depth				
M_1_–M_0_	0.37 ± 0.35	0.32 ± 0.31	0.640	(−0.08, 0.18)
M_3_–M_0_	0.32 ± 0.32	0.28 ± 0.29	0.718	(−0.08, 0.16)
M_6_–M_0_	0.13 ± 0.12	0.24 ± 0.26	0.040*	(−0.18, −0.02)
Grade of NLFs				
M1	1 (1,2)	1 (1,2)	0.820	(−0.29, 0.29)
M3	1 (1,2)	1 (1,2)	0.904	(−0.27, 0.24)
M6	2 (2,2.75)	2 (1,2)	0.013*	(−0.26, 0.26)

*Note:* All NLF dimension data are expressed as mean ± standard deviation and analyzed using the independent samples t‐test, with statistical significance set at *p* < 0.05. All Grade of NLFs data are presented using the median (P25, P75) and analyzed with the Mann–Whitney U test, with statistical significance set at *p* < 0.05.

Abbreviations: M_0_, pre‐treatment; M_1_, post‐treatment immediately; M_3_, post‐treatment 3 months; M_6_, post‐treatment 6 months.

* Statistical significance at *p* < 0.05.

Immediately post‐treatment & at 3 months: Reductions in all three dimensions were observed in both groups. However, the differences between collagen and HA were not statistically significant at these early time points.

At 6 months: A significant divergence in efficacy emerged, with HA demonstrating superior longevity.

Length change: Collagen group improved by 1.68 mm versus 2.67 mm for HA (*p* = 0.017).

Width change: Collagen group improved by 0.28 mm versus 0.49 mm for HA (*p* = 0.004).

Depth change: Collagen group improved by 0.13 mm versus 0.23 mm for HA (*p* = 0.040).

Clinical relevance: The significantly greater improvement in the HA group at 6 months indicates that, compared with the more persistent HA, collagen exhibited a faster rate of degradation and loss of effect.

### 3.3 NLFs Grading and Treatment Success (Table 2)

Both fillers effectively reduced the median NLFs grade from 3 to 1 immediately and at 3 months post‐treatment, with no inter‐group difference.

At the 6‐month follow‐up, the median grade for both groups had reverted to 2, but the distribution of scores was significantly different (*p* = 0.013), suggesting a variable pattern of effect maintenance.

All treatments were deemed successful based on the pre‐defined criteria.

### 3.4 Global Aesthetic Improvement Scale (GAIS) Scores

GAIS scores from both observers and patients are detailed in Table 3.

**TABLE 3:** GAIS of nasolabial folds post‐treatment.

GAIS	Experimental group	Control group	*p*	95% CI
M1				
Observer score	3 (2.25,3)	2 (2,3)	0.033*	(0.74, 1.26)
Patient score	3 (3,3)	3 (2,3)	0.429	(−0.21, 0.21)
M3				
Observer score	3 (2,3)	2 (2,3)	0.046*	(0.71, 1.29)
Patient score	3 (2,3)	3 (2,3)	0.602	(−0.29, 0.29)
M6				
Observer score	2 (1,2)	2 (2,2)	0.038*	(−0.21, 0.21)
Patient score	1.5 (1,2)	2 (2,3)	0.004*	(−0.29, 0.29)

*Note:* All data are presented using the median (P25, P75) and analyzed with the Mann–Whitney U test, with statistical significance set at *p* < 0.05.

* Statistical significance at *p* < 0.05.

Observer Assessment: Independent evaluators consistently rated the collagen group higher.

Scores were significantly better for collagen immediately post‐treatment (median: 3 vs. 2, *p* = 0.033) and at 3 months (median: 3 vs. 2, *p* = 0.046).

At 6 months, while the median score for both groups was 2, the observer GAIS remained significantly in favor of collagen (*p* = 0.038).

Clinical relevance: A GAIS score of three represents “Much Improved,” a threshold widely recognized as a marker of clinically meaningful aesthetic success. The collagen group maintained this superior rating longer.

Patient self‐assessment: Patients' self‐reported satisfaction was high and similar between groups at immediate and 3‐month follow‐ups (median score: 3). At 6 months, patient satisfaction was significantly higher in the HA group (median: 2 vs. 1.5, *p* = 0.004).

### 3.5 Adverse Events

The safety profile notably favored the collagen filler (Table 4).

**TABLE 4:** Complications post‐treatment.

Complications	Experimental group	Control group	*p*
Bruising	2	1	0.558
Swelling	0	6	0.012*
Nodule	0	1	0.312
Discoloration	0	3	0.078
Displacement	0	4	0.041*

* Statistical significance at *p* < 0.05.

Swelling: Occurred in 12% of the HA group but was absent (0%) in the collagen group (*p* < 0.05).

Filler displacement: Observed in 8% of the HA group and none (0%) in the collagen group (*p* < 0.05).

Other events: Incidences of discoloration were low and comparable between groups.

## 4 Cases Report

The following two cases illustrate the typical results commonly observed in our study.

### 4.1 Case 1

**FIGURE 3:**
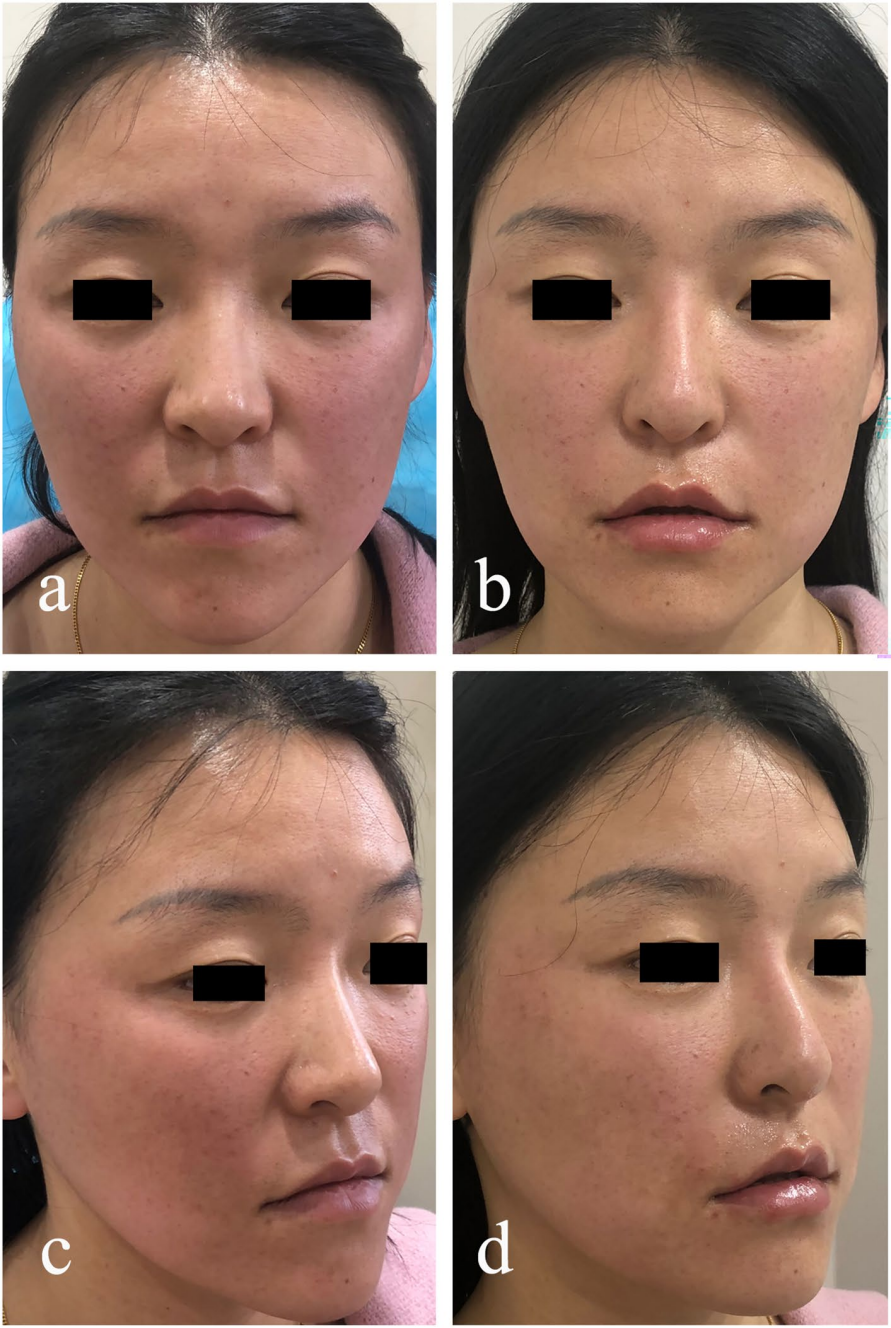
The patient in the experimental group, with a nasolabial fold aging grade of 3, demonstrated favorable outcomes following collagen injection.

This 31‐year‐old female patient was enrolled in the experimental group. The pretreatment assessment graded her NLFs as Class 3. The length, width, and depth of the left NLF measured 21.56, 5.42, and 0.88 mm, respectively; the corresponding dimensions of the right NLF were 18.64, 4.79, and 0.91 mm. A total of 3.5 mL of collagen was administered during the treatment. Immediately post‐treatment, the NLFs severity was improved to Class 1. The left NLF measurements were reduced to 13.11 mm in length, 3.27 mm in width, and 0.49 mm in depth, while the right NLF measured 11.17, 3.49, and 0.44 mm, respectively. The outcome was deemed clinically satisfactory upon immediate evaluation (Figure 3).

### 4.2 Case 2

**FIGURE 4:**
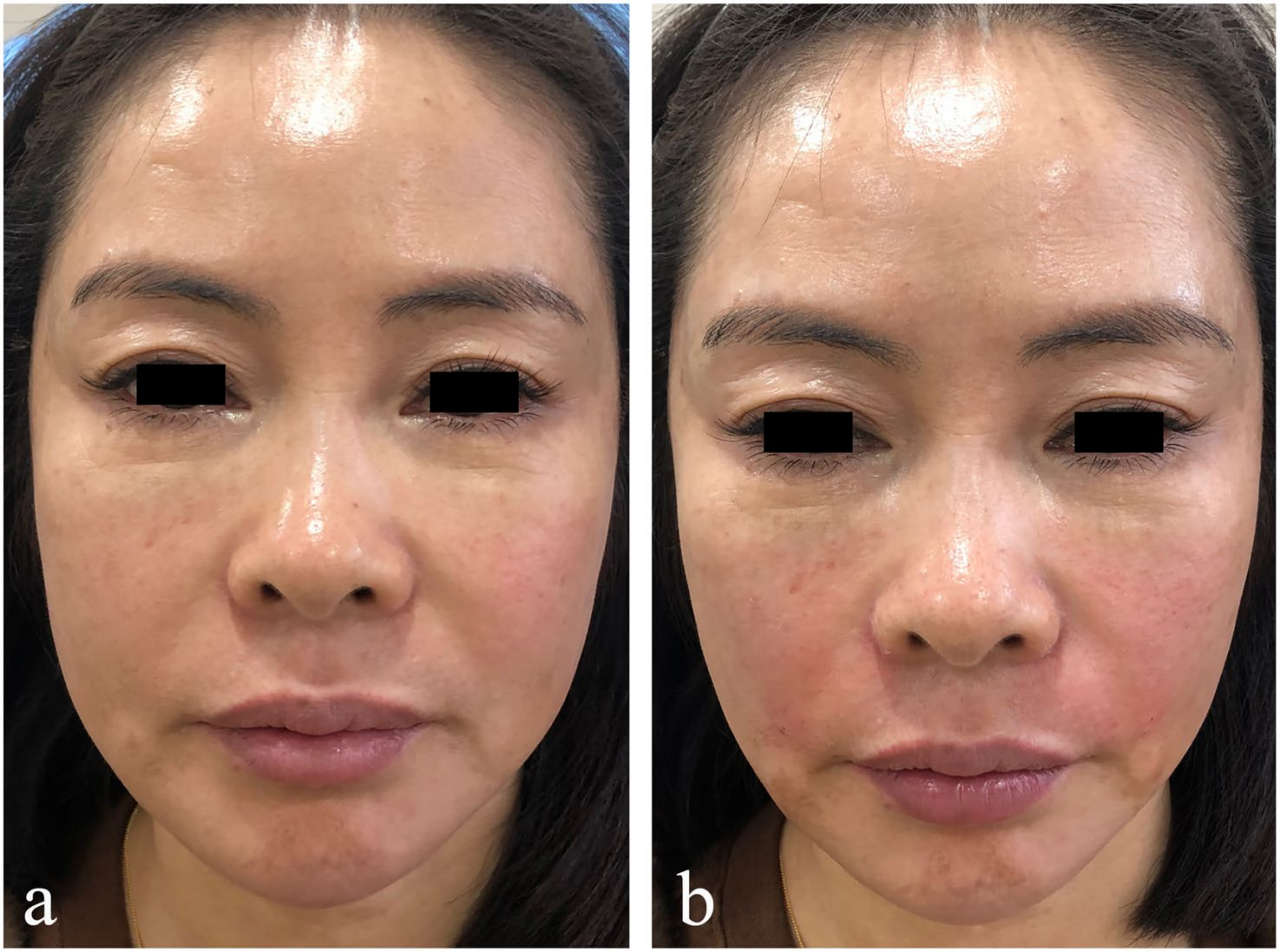
The patient in the control group, with a nasolabial fold aging grade of 3, exhibited effective improvement in nasolabial groove depression after hyaluronic acid injection, though mild swelling was observed.

This 42‐year‐old female patient was assigned to the control group. The pretreatment severity of her NLFs was graded as Class 3. The length, width, and depth of the left NLF measured 20.16, 6.42, and 0.83 mm, respectively, while those of the right NLF were 18.71, 5.79, and 0.74 mm. A total of 3 mL of HA was injected, which effectively augmented the depressed regions. Immediately after the treatment, the filler provided satisfactory correction, and the NLFs severity was improved to Class 2. The post‐treatment dimensions of the left NLF were 16.13 mm in length, 4.41 mm in width, and 0.63 mm in depth; those of the right NLF were 15.71, 3.89, and 0.52 mm, respectively. However, mild swelling was observed in the NLFs area due to the hydrophilic properties of HA (Figure 4).

## 5 Discussion

Our study demonstrated that both HA and collagen fillers provided comparable short‐term efficacy in reducing NLFs severity and dimensions immediately post‐treatment and at 3 months. Collagen yielded superior immediate and 3‐month aesthetic outcomes based on observer GAIS scores, while HA showed better durability in NLF dimensions and higher patient satisfaction at 6 months. Additionally, HA was associated with more adverse events, including swelling (12% vs. 0%) and filler displacement (8% vs. 0%). These trade‐offs highlight that treatment selection should depend on patient priorities, such as immediate results versus long‐term maintenance. These findings align with existing literature on dermal fillers. A 2024 prospective randomized trial reported durable NLF correction with a novel HA filler up to 9 months, consistent with HA's superior persistence observed here [10]. Similarly, a 2024 retrospective review noted higher rates of edema and displacement with HA, mirroring our safety profile [11]. Comparative studies in Asian populations further emphasize variations in filler efficacy and safety, underscoring the need for ethnicity‐specific research on collagen [12].

The divergent outcomes can be attributed to rheological differences: collagen's lower G' and viscosity enable rapid tissue integration for natural immediate results but lead to faster degradation under dynamic facial forces. In contrast, HA's higher G' and cohesivity (e.g., in Restylane Lyft) provide sustained structural support, enhancing long‐term persistence [13]. HA's hydrophilic properties also explain post‐injection swelling, peaking at 24–72 h and resolving in 3–7 days, while migration in the perioral area typically lasts 1–2 weeks and can be managed with massage or hyaluronidase [14].

Clinically, collagen is ideal for younger, socially active patients seeking rapid enhancements before events, accepting shorter duration and repeat treatments despite budget considerations. HA suits older patients with severe aging, prioritizing longevity, safety, and fewer follow‐ups, even with transient side effects. Selection should integrate age, lifestyle, budget, and setting—favoring collagen in outcome‐focused private practices and HA in safety‐oriented public hospitals—for personalized optimization.

This study has several limitations. Manual NLF measurements introduce subjective bias, particularly for depth; future work should employ 3D scanners or laser profilometers for objectivity. The 6‐month follow‐up is short, limiting long‐term insights; extensions to 9–12 months would clarify durability. The small sample size (*n* = 100) and single‐center design restrict statistical power and generalizability across ethnicities and skin types.

Future directions include adopting 3D imaging for precise assessments and conducting multicenter trials to enhance result applicability across diverse populations.

## 6 Conclusion

This prospective comparative study revealed comparable short‐term efficacy between HA and collagen fillers for NLFs correction immediately post‐treatment and at 3 months. Collagen provided superior immediate aesthetic outcomes, while HA demonstrated greater durability at 6 months. Collagen fillers offer excellent immediate aesthetic improvement with minimal downtime, whereas HA fillers provide superior persistence at 6 months. Treatment choice should be individualized based on desired onset, duration, cost, and tolerance for mild transient swelling. Both are safe and effective, with distinct roles depending on patient factors; further validation through larger samples and extended follow‐up (e.g., 9–12 months) is recommended.

## Author Contributions

C.H. and J.L. performed the research. B.S. and Q.L. supervised the research study. L.G. and C.H. analyzed the data. J.L. and C.H. wrote the paper.

## Funding

The authors have nothing to report.

## Ethics Statement

This study was conducted following the principles of the Declaration of Helsinki and was approved by the local ethics committee (KY20252351‐C‐1). Written informed consent was obtained from all participants, and all privacy and data protection regulations were strictly followed. As a prospective randomized controlled trial, it was registered with the Chinese Clinical Trial Registry.

## Consent

Written informed consent was obtained from the individual depicted in this photograph for the use of their likeness in this publication. They have explicitly agreed to the use of their image in both print and online formats of the journal, as well as in derivative works, and understand that their identity may be apparent.

## Conflicts of Interest

The authors declare no conflicts of interest.

## Data Availability

The data that support the findings of this study are available from the corresponding author upon reasonable request.
